# 

**Published:** 1907-05

**Authors:** 


### The American Journal of Dental Science

The Official Organ of the Florida, Georgia and South Carolina State Dental Societies

Third Series

VOL. XXXVIII MAY, 1907 NO. 5

EDITORS

WM. GIRD BEECROFT, D. D. S., Madison. Wis. B. H. TEAGUE, D. D. S., Aiken, S. C.

Published on the 10th day of each month.
Subscription price $1.00 per year.
ENTERED AS SECOND-CLASS MATTER, MADISON, WIS.

Address all communications, both business and editorial, to
The Dental Publishing Company, Madison, Wisconsin

### TRANSPORTATION RATES TO THE JAMESTOWN DENTAL CONVENTION.

The following rates to the Exposition have been made by the transportation lines: Season tickets, 80 per cent. of double one way. Sixty day tickets one and one-third fare plus 25 cents. Ten day tickets one and one-third fare plus $2.25.

These rates will probably be lessened, or, if not, there are likely to be special excursions from all parts of the country and Canada at low rates.

The following places of interest can be visited as side trips:

Jamestown Island, $1.00; Yorktown, $1.00; Williamsburg, $1.95; Washington, $3.50; Baltimore, $5.00; New York, Old Dominion SS. Co., $13.00 round trip; Philadelphia, rail, $9.00 round trip; Richmond, $3.50 round trip.

### MEETINGS.

The thirty-seventh annual convention of the South Carolina State Dental Association will be held in the city of Anderson, S. C., commencing July 2d and continuing through 3d, 4th and 5th. Special hotel rates have been secured, also one and one-third railroad rates on the certificate plan. All ethical practitioners are most cordially invited to attend.—Euston N. Kibler, cor. sec'y, Prosperity, S. C.

Several watering places within a few minutes ride of Norfolk and Exposition ground. For further information address, Committee on Transportation, Jamestown Dental Convention, J. Lewis Walker, A. Allison Stores, W. M. Sturgis, Norfolk, Va.

The Virginia State Dental Association will hold its annual meeting the 9th of September, 1907, at the Inside Inn, Jamestown Exposition. There will only be a short session as the activities of our members are being merged with those of the Jamestown Dental Convention. This will be strictly a business meeting. No committees will be appointed; and no work done other than certain important matters of business which will be designated later in a circular letter to be issued to each member.—W. H. Piarson, ass't cor. secretary.

### INTERSTATE DENTAL FRATERNITY.

The board of governors of the Interstate Dental Fraternity will convene for the annual business meeting of the order in Minneapolis, Minn., Monday, July 29, at the West Hotel. The annual banquet will occur during the week and due notice thereof will be sent to the members as soon as arrangements can be made and the exact date fixed. It is hoped that the fraternity will meet in large numbers on this occasion. Dr. R. M. Sanger, National Secretary, East Orange, New Jersey.

